# Synthetic xylan-binding modules for mapping of pulp fibres and wood sections

**DOI:** 10.1186/1471-2229-7-54

**Published:** 2007-10-12

**Authors:** Lada Filonova, Lavinia Cicortas Gunnarsson, Geoffrey Daniel, Mats Ohlin

**Affiliations:** 1 WURC, Department of Wood Science, Swedish University of Agricultural Sciences, PO Box 7008, SE-750 07 Uppsala, Sweden; 2 Department of Immunotechnology, Lund University, BMC D13, S-22184 Lund, Sweden; 3 Current address : Affitech AS, Oslo, Norway

## Abstract

**Background:**

The complex carbohydrate composition of natural and refined plant material is not known in detail but a matter that is of both basic and applied importance. Qualitative assessment of complex samples like plant and wood tissues requires the availability of a range of specific probes. Monoclonal antibodies and naturally existing carbohydrate binding modules (CBMs) have been used in the past to assess the presence of certain carbohydrates in plant tissues. However, the number of natural CBMs is limited and development of carbohydrate-specific antibodies is not always straightforward. We envisage the use of sets of very similar proteins specific for defined targets, like those developed by molecular evolution of a single CBM scaffold, as a suitable strategy to assess carbohydrate composition. An advantage of using synthetic CBMs lies in the possibility to study fine details of carbohydrate composition within non-uniform substrates like plant cell walls as made possible through minor differences in CBM specificity of the variety of binders that can be developed by genetic engineering.

**Results:**

A panel of synthetic xylan-binding CBMs, previously selected from a molecular library based on the scaffold of CBM4-2 from xylanase Xyn10A of *Rhodothermus marinus*, was used in this study. The wild type CBM4-2 and evolved modules both showed binding to wood sections. However, differences were observed in the staining patterns suggesting that these modules have different xylan-binding properties. Also the staining stability varied between the CBMs, the most stable staining being obtained with one (X-2) of the synthetic modules. Treatment of wood materials resulted in altered signal intensities, thereby also demonstrating the potential application of engineered CBMs as analytical tools for quality assessment of diverse plant material processes.

**Conclusion:**

In this study we have demonstrated the usefulness of synthetic xylan-binding modules as specific probes in analysis of hemicelluloses (xylan) in wood and fibre materials.

## Background

Wood cell walls represent highly complex biocomposites that consists of repertoires of polysaccharides and lignins integrated into a three-dimensional conglomerate [1,2]. At the microstructural level, wood cell walls are composed of both primary- and dominating secondary cell wall layers in which the major (cellulose, lignin and hemicelluloses) and minor (pectins, proteins) chemical components are heterogeneously dispersed and integrated. Understanding the cell wall's complexity is of great interest both for plant biology and for plant processing in food and pulp industry.

One of the key main challenges for an improved understanding of wood cell wall structure especially, in relation to properties, is an ability to specifically localise and visualised the spatial distribution of the major polysaccharide components (cellulose and hemicelluloses) *in vivo*. In lignified cell walls this is quite challenging since the hemicelluloses interface between the cellulose and lignin forming a heterogeneous polymer complex and it is exacting to distinguish between the various components in the different cell wall layers. Xylan is the dominant hemicellulose found in hardwoods and depending on species, normally represents 15–30% of the total dry matter content [3]; the major component being an O-acetyl-4-O-methylglucurono-β-D-xylan referred as glucuronoxylan. In softwoods, xylan normally forms 5–10% of the total dry matter as arabinoglucuronxylan and consists of a backbone of 1,4-linked β-D-xylopyranose units, partially substituted by 4-O-methyl-α-D-glucuronic acid and α-L-arabinofuranose units [3]. Knowledge on the microdistribution of hemicelluloses (i.e. xylans) within wood cell walls has come primarily from early gross chemical studies on sections from cambium layers at different stages of cell wall differentiation [4], by the application of xylanase-gold complexes to plant cell walls and pulp fibres [5-7] and by microscope observations after selective xylan removal [8,9]. More recently monoclonal antibodies specific for xylans have been successfully used as probes on plant fibres (flax) and other non-lignified plant materials [10], but not wood. There has also been great interest to chemically map and assess the removal and sorption of xylans onto fibres during kraft pulping processes in order to develop improved washing and preventive methods thus improving the technical properties of paper products [5]. However, apart from these examples very little is known on the microdistribution of xylans across lignified wood cell walls.

A plethora of direct (e.g. enzyme-gold) and indirect methods (e.g. antibodies) have been employed to visualise carbohydrates in plant materials by both light- and electron microscopy [11,12]. Historically, antibodies have been the markers of choice and a range of monoclonal probes have been produced for visualising plant cell wall carbohydrates (e.g. xylans, galactans, glucomanans, arabinans and fucosylated xyloglucan) [10,13,14]. More recently, several non-catalytic carbohydrate binding modules (CBMs) have also been reported to show great potential as specific and versatile markers of carbohydrates in plant materials [15-19]. CBMs are a group of natural binders that most often constitute parts of modular glycoside hydrolysing or modifying enzymes. Based on their sequences, CBMs are classified into 49 families http://http:afmb.cnrs-mrs.fr/CAZY/, however, there are large variations in binding specificities, towards crystalline, amorphous and soluble polysaccharides, both between and within the families [20,21]. CBMs represent an attractive alternative to antibody probes not only because of their size (e.g. typically < 20 kDa vs ~150 kDa for IgGs) and intrinsic specificity to individual carbohydrates – often different regions of the same substrate may be targeted- but also because of their ease of production and ability for modification with peptides and fluorescent (e.g. fluorescein isothiocyanate (FITC), tetramethylrhodamine isothiocyanate (TRITC)) and gold markers for microscopy [15-17,22-24]. We have previously developed methods for detection of cellulose in wood cell walls by visualising native cellulose-specific CBMs (i.e. CBM_*PcCel*7*D*_, CBM1_*HjCel*7*A*_) bound to their target in both light- and electron microscopy [15-17]. Although several natural CBMs are available for these applications, the numbers are relatively low. It is reasonable to expect that probes showing superior performance in given applications will be available by having a larger population of molecules to choose from. Combinatorial library and selection technologies provide a solution to this need. Here we further developed this approach and report on the application of a range of engineered xylan-binding CBMs for targeting xylan in wood (birch and pine) sections and on the surface of birch kraft pulp fibres. The advantages of using synthetic CBMs -which is novel for this study- over native CBMs is related to their molecular properties such as stability and binding specificity that can be targeted by engineering. The synthetic xylan-binding CBMs utilised in this work for *in-situ *visualisation of xylan in fresh wood sections and whole fibre materials performed well, clearly demonstrating their usefulness in studies on plant materials.

## Results and discussion

### Binding properties of xylan-binding CBMs

**Figure 1:**
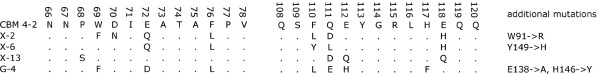
**Sequence differences between the five different modules**. Each protein is 165 amino acid residues long not counting the hexa-histidine tag, used in this investigation. Amino acids are represented by the standard one-letter code. Identities with the wt CBM4-2 sequence are shown by dots. Residue numbering is in accordance with Simpson et al. [25].

**Figure 2:**
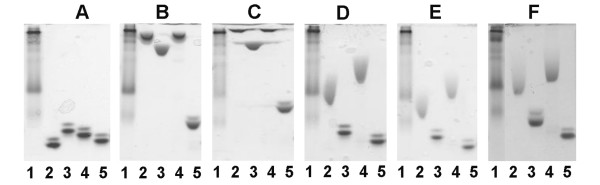
**Affinity electrophoresis of soluble CBM-variants**. Affinity electrophoresis performed in the absence of a ligand (A) or in the presence of 0.2% (w/v) oat spelt xylan (B), 0.1% (w/v) arabinoxylan (C), 0.05% (w/v) barley β-glucan (D), 0.1% (w/v) lichenan (E) or 0.05% (w/v) non-fucosylated xyloglucan (F). Samples included: lane 1, Kaleidoscope prestained standard; lane 2, wt CBM4-2; lane 3, X-2; lane 4, X-6; lane 5, X-13.

The wild type (wt) CBM4-2 from the xylanase Xyn 10A of *Rhodothermis marinus *is a type B CBM [20] displaying a groove-shaped binding site that fits polysaccharide chains well [25]. This CBM binds not only to xylan but also other non-crystalline plant carbohydrates [26,27]. In a previous study, we had constructed a combinatorial library of CBM4-2 [28] and used the phage-display system to select for CBM-variants specific for different ligands. The binding properties of three CBMs, X-2, X-6 and X-13, all selected using the insoluble part of birch wood xylan as a selection target were further investigated in this work with the aim of defining the potential to use such modules in analysis of xylans in plant tissues. The sequences of these proteins and a control module unable to bind plant carbohydrates are all very similar and they differ from the wt protein (of 167 residues) by only 4–9 amino acid substitutions (Figure 1). Despite the fact that they originate from the same selection, these CBMs have different affinities and specificities for a number of tested carbohydrates. Affinity electrophoresis demonstrated that the X-6 variant, like wt CBM4-2, binds to a range of carbohydrates (Figure 2). In contrast, both X-2 and X-13 are substantially more specific for xylans and do not recognise xyloglucan or glucan-containing carbohydrates (Figure 2). In addition, isothermal titration calorimetry studies further confirmed that X-2 was much more prone to bind xylans than any other carbohydrate targets investigated further demonstrating its modified specificity [27]. Relative binding affinities, determined by affinity electrophoresis, revealed a decrease in affinity for xylan of X-2 and X-13 and a somewhat higher affinity for xylan of X-6 compared to the wt CBM4-2 (Table 1). Altogether we have demonstrated our access to a range of very sequence-similar CBMs with diverse binding properties that can be used as a utility in analysis of the carbohydrate composition in lignocellulose materials.

**Table 1:** Affinities of CBM-variants for birch wood xylanas determined by affinity electrophoresis

**Protein**	**K_D _(mg/ml)**	**Relative affinity (%)**
wt CBM4-2	0.12	100
X-2	0.22	52
X-6	0.07	157
X-13	1.00	12

### Binding of the CBMs to untreated wood sections

In order to assess the ability of the xylan-binding modules to function as analytical probes, we used these proteins for staining tissue samples derived from three wood species known to differ in their content of xylan. A very high content of xylan-based carbohydrates is characteristic for *Betula verrucosa *(birch) wood [29,30]. *Pinus sylvestris *(pine) wood contains approximately 20% of the xylan found in birch wood [31,32], while wood derived from *Populus tremuloides *(poplar) is intermediate [33-35]. Moreover birch and poplar tension wood, but not pine wood, contains fibres with a special morphology and chemical composition due to development of a gelatinous layer (G-layer) [36,37]. The G-layer is known to have high cellulose content with a high degree of crystallinity [34,38,39]. We assume that the combination of almost pure cellulose in the G-layer and hemicelluloses in S1, S2 and S3 layers make tension wood an interesting model for assessment of carbohydrate specific probes. We therefore assessed the xylan-binding CBMs that displayed different xylan-binding properties, as outlined above in these models.

**Figure 3:**
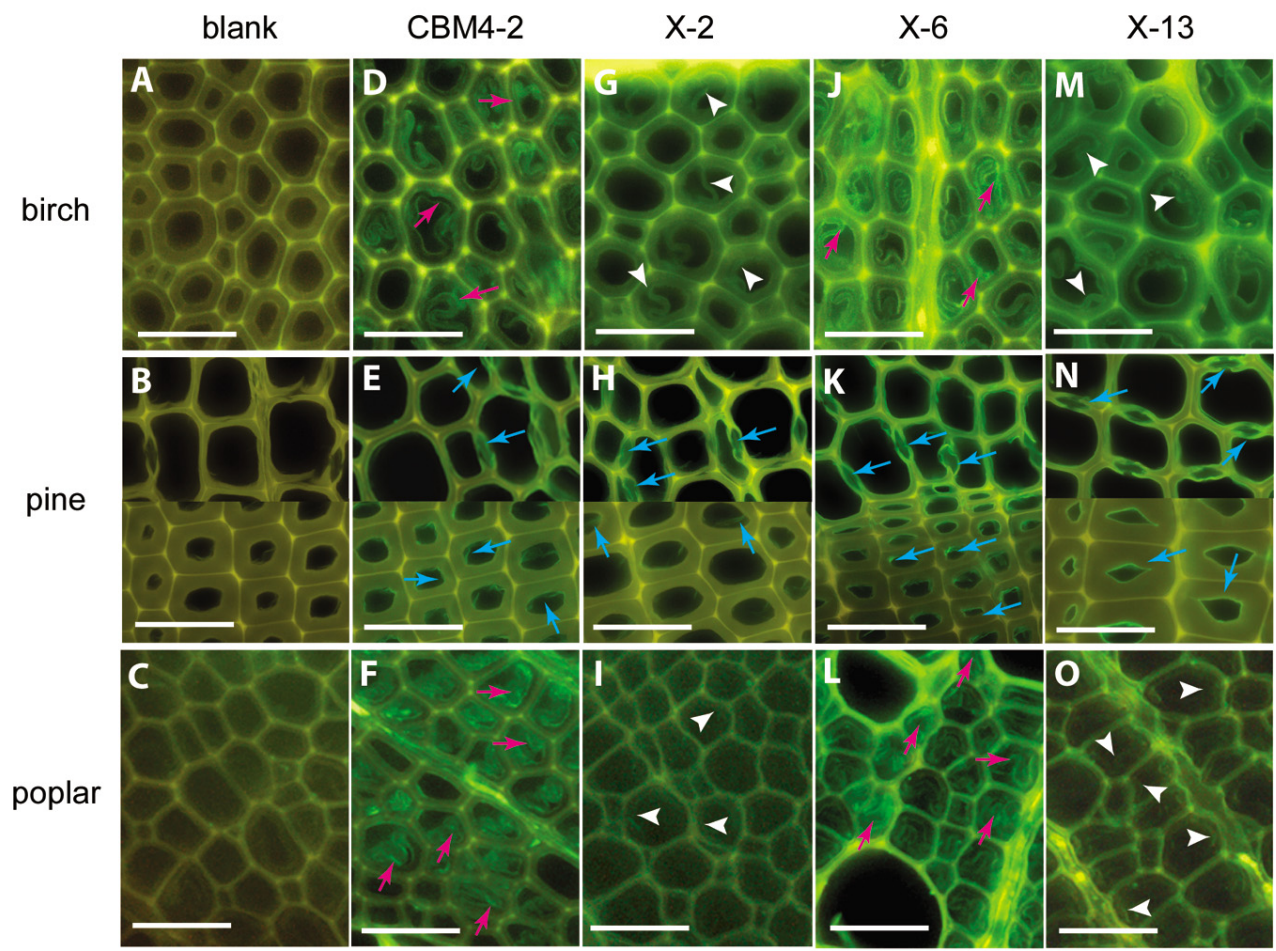
**Binding of CBMs to different wood sections**. Binding of wt CBM4-2 (D-F), X-2 (G-I), X-6 (J-L) and X-13 (M-O) to birch, pine and poplar wood sections. Negative control staining of wood samples with mouse anti-His antibody and FITC-conjugated anti-mouse IgG is shown in (A-C). All modules bind to border pit chambers in earlywood and to the latewood lumen wall in pine (blue arrows). Pink arrows show staining of G-layers with wt CBM4-2 and X-6 in birch and poplar cell walls (D, F, J, L), a staining that was not observed with X-2 and X-13 (G, I, M, O; white arrowheads). Note all images are from transverse sections and images of pine sections include examples of both early- (above) and latewood (below). Scale bar = 50 μm.

Fluorescent microscopy demonstrated substantial differences in staining patterns of birch, poplar and pine wood sections with wt CBM4-2, X-2, X-6 and X-13 (Figure 3). All CBMs showed binding in comparison to the control samples (Figure 3A – C), wt CBM4-2 and X-13 displaying the most efficient wood labelling (Figure 3D – F; M – O). Although less intense, more stable staining was characteristic for X-2 staining experiments (Figure 3G – I), where the same intensity of fluorescent signal was retained for at least 72 hours giving this module an important advantage as a specific binding probe in assay setups that demand higher labelling stability. The observed (Figure 2, Table 1) reduced affinity for xylan of some of the engineered modules, in particular X-13, obviously did not prevent their use in these applications. The binding of lower affinity CBMs is likely to be facilitated by high local concentration of repetitive structures recognised by them in plant tissues. Alternatively, the affinity of e.g. X-13 for some specific structures in xylan may be higher than that observed when assessing the affinity for solubilized carbohydrates allowing for its use in staining of plant materials.

In birch and poplar samples, wt CBM4-2 and X-6 bound to the cell wall but predominantly to G-layers reported to consist of almost pure cellulose (Figure 3D, F, J, L) whereas X-2 and X-13 signals were detected within S1, S2 layers and middle lamellar regions (Figure 3G, I, M, O). These differential staining patterns are best explained by different affinity and probably more importantly specificity profiles of the CBMs, as outlined above. Given the inability of these modules to bind crystalline cellulose, it is possible that wt CBM4-2 and X-6 CBMs labelled xyloglucans within the G-layer, possibly in areas with residual lignin [40]. To define in more detail the presence of binding sites recognised by these xylan-binding CBMs, it was observed that all CBMs, *i.e*. both those that are (wt CBM4-2 and X-6) and those that are not (X-2 and X-13) cross-reactive to other types of hemicellulose carbohydrates like xyloglucans, stained pine samples in areas of bordered pit chambers (including torus) in earlywood and on the inner surface of cells in latewood (Figure 3E, H, K and 3N). There is however no previous evidence for the existence of xyloglucans in bordered pit chambers. It has been shown previously using selective enzyme removal and enzyme-antibody labelling that bordered pit membranes in pine sapwood tracheids are composed of non-lignified pectin and a cellulose-rich region in the centre of the pit called a torus, which is supported by a surrounding margo that contains microfibrils of cellulose [41,42]. The fact that modules that are quite specific for xylose oligomers bind to these structures, suggests presence of at least rudimentary xylan in pine bordered pit chambers.

Variation in binding patterns of the different CBM-variants to xylan embedded in the wood cell walls was also observed in the study of McCartney et al. [18] where some natural xylan-binding CBMs stained both primary and secondary cell walls while some (including CBM4-2) only stained secondary walls. Apart from the CBM's specificity and the actual presence of particular xylan structures, ligand accessibility is also a determinant factor for specific staining of plant materials using this type of modules. The binding mechanism of many xylan-binding CBMs involves sandwiching of the ligand, which then requires access to both faces of the carbohydrate chain that may be a limitation in lignocellulose materials. It is believed that the X-2 module has evolved to use only one aromatic residue for interaction with only one face of the xylan chain [27]. These binding features of X-2 could facilitate its binding to the embedded xylan chains and may explain the very stable X-2 staining in the present study. Altogether, the availability of following a molecular evolution procedure of sets of CBMs with defined binding properties, some members of which are less cross-reactive in comparison to naturally occurring CBMs [27], allow assessment and profiling of carbohydrate composition of plant cell walls and define differences observable between various samples.

### Binding of the CBMs to treated wood sections and pulp fibres

As outlined above, CBMs derived from the library built on the CBM4-2 scaffold [28] can be used to investigate native and normal wood tissues. Modification of such material by different means may add additional information concerning their construction. To exploit these possibilities, we investigated the ability of X-2 to recognise xylan molecules in birch and pine wood samples modified through biotechnological processing.

**Figure 4:**
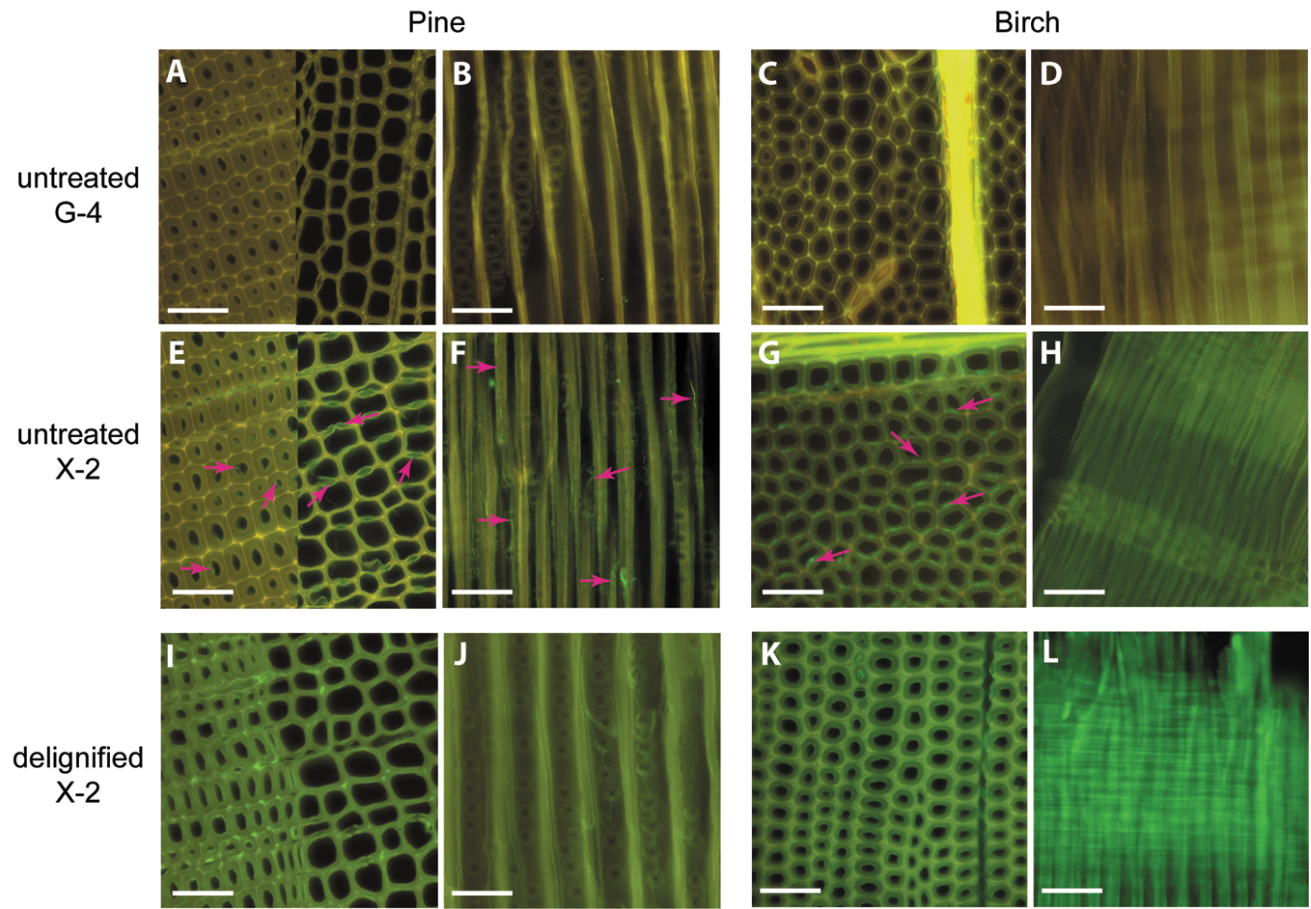
**Binding of FITC-conjugated X-2 to wood samples**. Pine and birch sections stained with either FITC-conjugated G-4 (A-D) (negative control) or with FITC-conjugated X-2 (E-L). Sections were either untreated (A-H) or delignified (I-L) prior to staining. Pink arrows show signals in untreated samples. Note that A, C, E, G, I and K are from transverse sections and B, D, F, H, J and L are from longitudinal sections. Pine sections in A and E show both early- (to the left) and latewood (to the right). Scale bar = 50 μm.

**Figure 5:**
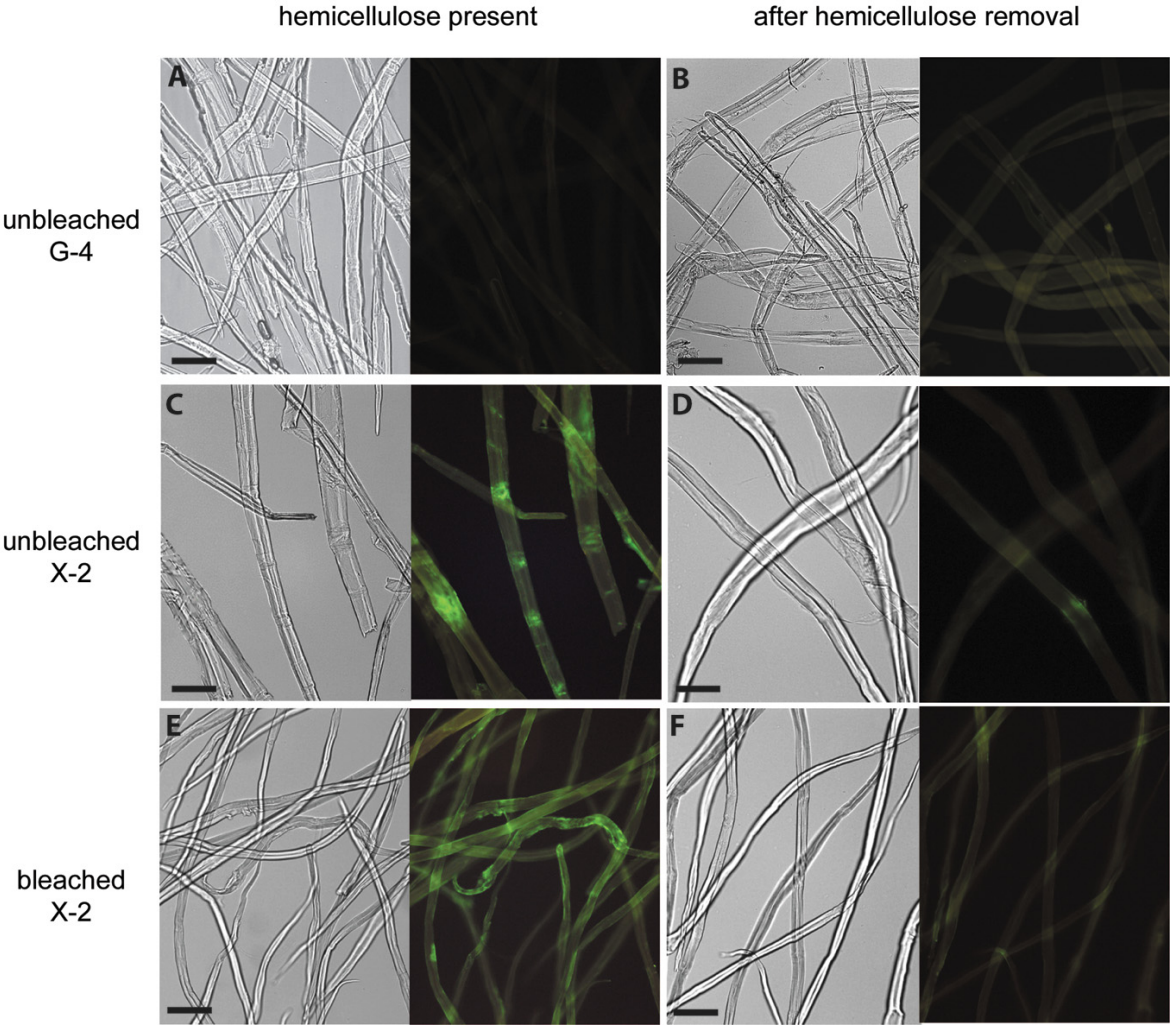
**Binding of FITC-conjugated X-2 to birch pulp**. Control staining of fibres with FITC-conjugated G-4 is shown in A (unbleached birch pulp) and B (unbleached birch pulp, after hemicelluloses removal). Binding of X-2 to unbleached pulp fibres was mostly observed at the sites of fibre damage (C), whereas bleached birch pulp were more uniformly stained (E) like the wood sections in Figure 4. Removal of hemicelluloses from both pulp types reduced the fluorescent signal (D, F) proving the specificity of X-2 for xylan. It was possible to treat pulps until complete removal of xylan recognised by X-2 was obtained. Scale bars = 100 mm for A – D, 200 mm for E, F.

Staining of delignified tissue samples (Figure 4I–L) with FITC-conjugated X-2 resulted in much higher signals in comparison to staining of mature (normal) birch and pine tissues (Figure 4E – H). In normal pine sections, X-2 binding was mainly detected in border pit regions (earlywood) and inner surface of cells (latewood) (Figure 4E, F) whereas in birch section staining was observed mostly in middle lamellae regions (Figure 4G, H). In contrast, signals from X-2 binding were evenly distributed over the surfaces of delignified sections in both wood types (Figure 4I – L). This finding suggests, as expected, that removal of lignin increases the access to the xylan binding sites. Staining of delignified samples by various CBMs may be beneficially preformed to ensure the efficiency of the delignification procedure and to exploit the access of specific carbohydrate epitopes prior to enzyme treatment (high cost procedures) of wood materials. Similarly, hemicellulose removal following a bleaching procedure could be efficiently assessed in pulp fibres (Figure 5) further expanding the area of useful applications of these molecularly engineered modules.

## Conclusion

In this study, we have demonstrated that molecularly engineered CBMs based on the CBM4-2 scaffold can have a wide range of applications in basic wood research. We envisage that such engineered modules will provide the research community with powerful new tools to complement existing detection reagents such as monoclonal antibodies or natural CBMs. In addition to the xylan-specific CBMs used in this work, we have also reported the successful selection of CBM-variants from the same molecular library for their specificities towards other plant cell wall carbohydrates, such as mannan and xyloglucan [28,43]. The use of engineered CBMs in future studies will further expand the potential of these types of synthetic probes for mapping of pulp fibre and wood sections. The binding properties of these modules would also make them useful in the analysis of technological processes including those relevant for quality assessment of industrial processes.

## Methods

### Production and purification of CBMs

Protein variants evolved from the scaffold of CBM4-2, from the xylanase 10A of *Rhodothermus marinus*, were selected in a previous study [28] for binding to birch wood xylan or binding to a human IgG4 molecule. Three CBM-variants from the selection towards xylan (X-2, X-6 and X-13), the wt CBM4-2 and a human IgG4-specific module (G-4) that served as a non-carbohydrate binding control for the scaffold, were used in this work. Genes coding for the proteins with a C-terminal His_6_-tag had previously been cloned in the pET-22b(+) vector (Novagen, Madison, WI, USA) and then transformed in the *Escherichia coli *strain BL21(DE3) (Novagen). These genes were now expressed and the soluble CBMs were purified using metal-ion-affinity chromatography as described earlier [26,28].

### FITC-labelling of CBMs

One of the xylan-specific CBMs, X-2, and the control module G-4 were labelled with fluorochrome. This labelling is facilitated by the fact that these, as well as most other variants selected from this combinatorial library, carries only two primary amino groups, both of which are located well away from the carbohydrate binding surface. FITC was dissolved in dried N, N-dimethyl formamide (DMF) to a concentration of 1 mg/ml. The purified CBMs, stored in 20 mM NaH_2_PO_4 _(pH 7.5), were first diluted 1:1 in 0.2 M Na_2_CO_3 _(pH 9) and FITC was slowly added to reach 10-fold molar excess relative to CBM. The reaction mixture was gently stirred in a glass vial for 1 hour at room temperature and then at 4°C overnight. NH_4_Cl was added to a final concentration of 50 mM in order to inactivate the un-reacted FITC molecules and stirring was continued for 3 hours. CBM-FITC conjugates were separated from FITC by using PD-10 desalting columns (Amersham Bioscience, Uppsala, Sweden) and eluting with 20 mM NaH_2_PO_4 _(pH 7.5). Several fractions were collected during elution and those with the highest absorbance at 280 nm were pooled together. The actual labelling ratios 1.6 (X-2) and 0.6 (G-4) mol fluorochrome/mol protein were determined spectrophotometrically by measuring the absorbance at 280 nm and 495 nm and using the extinction coefficient 195 for FITC at 495 nm.

### Affinity electrophoresis

Affinity electrophoresis was performed using the Bio-Rad (Hercules, CA, USA) mini-gel system as previously described [26]. Purified CBMs (3 μg per gel) were separated at room temperature at 90 V on native gels, containing none or different concentrations of oat spelt xylan (Sigma-Aldrich, St. Louis, MO, USA), birch wood xylan (Roth, Karlsruhe, Germany), arabinoxylan (Megazyme, Bray, Ireland), barley β-glucan (Megazyme), lichenan (Sigma-Aldrich) and tamarind seed xyloglucan (Megazyme). A kaleidoscope pre-stained standard (Bio-Rad) was included in each gel and proteins were detected by staining with Coomassie Brilliant blue or Simply Blue Safe stain (Invitrogen, Paisley, UK). Affinity constants for birch wood xylan were calculated according to the theory of affinity electrophoresis as described by Takeo [44] after performing a series of runs on gels with a ligand concentration ranging from 0.2 to 2 g/l. The relative mobility of a CBM-variant, r (in presence of ligand) and R (in absence of ligand), versus BSA used as a negative, non-interacting control were calculated from the migration distances, the distances between the major protein band and the migration front of the gel. The -1/K_D _value was obtained as the x-intercept of a straight line in a plot of 1/(R-r) versus 1/c according to the relationship:

1/(R-r) = (1 + K_D_/c)/R

where c is the ligand concentration in the gel.

### Preparation of plant materials

Serial wood sections (15 μm) from poplar (*Populus tremuloides*), birch (*Betula verrucosa Ehrh*.) and pine (*Pinus sylvestris L*.) were prepared using a Leitz microtome. Mild delignification was in some cases performed on semi-thin sections of pine and birch using a 1:1 (v/v) mixture of H_2_O_2 _and concentrated acetic acid prior to labelling. Alternate sections from the same wood specimens were used for each labelling experiment.

Oxygen bleached and unbleached birch pulp fibres (kindly provided by Eka Chemicals) both with and without hemicellulose removal (Table 2) were used for binding experiments with CBM-FITC. Hemicellulose removal was performed by treatment of pulp with 24% (w/v) potassium hydrochloride at room temperature for 6 h (shaking) followed by triple washing (20 min. each, shaking). Thereafter pulps were treated with 17.5% sodium hydrochloride (w/v) containing 4% (w/v) boric acid at room temperature for overnight.

**Table 2:** Characteristics of the birch pulps used for the labelling experiments

**Analyses**	**Unbleached**	**Standard ECF**	**A-ECF-Bleached**
Brightness [% ISO]	47.9	89.7	89.7
Viscosity [ml/g]	1085	977	969
Kappa Number	13.6	0.74	0.78
Total charge [mmol/kg]	132	67	63
Hemicellulose [% ]	27.65	26.3	26.5

### Binding of xylan-binding CBMs to wood sections and pulp fibres

Binding experiments on wood sections using all four xylan-binding CBMs (wt CBM4-2, X-2, X-6 and X-13) were performed as follows. An initial blocking step was carried out, for preventing any non-specific binding of CBMs, by incubating all samples in phosphate-buffered saline (PBS) (pH 7.4) containing 5% (w/v) ovalbumin. Wood samples (2 alternate sections per CBM-variant) were then incubated in 500 μl of PBS containing 10 μM CBM at room temperature for 6 h. Thereafter the samples were washed twice with PBS (5 minutes each step) and incubated with mouse Anti-His antibody (Amersham) at 4°C overnight. After washing three times with PBS (10 minutes each step), samples were treated with FITC-conjugated anti-mouse IgG (Sigma) diluted 1:500 in PBS for 1 h in room temperature. As negative control, wood sections were incubated with both antibodies.

In addition, wood sections, untreated or delignified, as well as bleached or unbleached birch pulp fibres (a constant dry weight of 2 mg) were incubated in 500 μl of PBS containing 2 μM FITC-conjugated X-2 and G-4 for 4 h at room temperature followed by washing in PBS. Since G-4, a module selected from the same library as the xylan-binding CBMs, had been shown unable to bind xylan and other hemicelluloses [28], its FITC-labelled derivative served as a negative control in these experiments.

In order to detect the bound CBM molecules (FITC-conjugated or by using labelled antibodies), the samples were placed on object glasses mounted in Fluorsave (Calbiochem, CA, USA), covered by cover slips and examined by fluorescent microscopy (Leica, Germany) using a standard set of filters for FITC. Images were recorded via a CCD camera (Leica DC 300F, Microsystem) using a digital imaging system for professional microscopy (Leica Microsystem Ltd, 2001) with one exposure time (2 s) for all samples in order to detect differences in signal intensity.

### Gene sequences

Sequences of protein modules used in this work are available from public databases as follows: CBM4-2: [GenBank:AAT06926], X-2: [GenBank:AAT06999], X-6: [GenBank:AAT07002], X-13: [GenBank:AAT07003] and G-4: [GenBank:AAT07011].

## Abbreviations

CBM, carbohydrate binding module; FITC, fluorescein isothiocyanate; PBS, phosphate-buffered saline; wt, wild type

## Competing interests

The author(s) declares that there are no competing interests.

## Authors' contributions

LF carried out the bio-analytical study involving the fluorescence microscopy and helped to draft the manuscript. LCG carried out the characterisation of the previously engineered proteins as well as FITC-labelling of the proteins and helped to draft the manuscript. GD participated in the coordination and design of the bio-analytical study and helped to draft the manuscript. MO participated in the coordination and design of the molecular engineering and protein characterisation studies and helped to draft the manuscript. All authors read and approved the final manuscript.
